# Beyond Ulcerations: A Case of Cutaneous Polyarteritis Nodosa in a Middle-Aged Woman

**DOI:** 10.7759/cureus.82840

**Published:** 2025-04-23

**Authors:** Saadia Boughaleb, Hanane Baybay, Imane Fadlallah, Layla Tahiri Elousrouti, FatimaZahra Mernissi

**Affiliations:** 1 Dermatology, Hassan II University Hospital, Fez, MAR; 2 Pathology, Hassan II University Hospital, Fez, MAR

**Keywords:** cutaneous polyarteritis nodosa, necrotizing vasculitis, pathology, polyarteritis nodosa, systemic vasculitis, ulcerated nodules

## Abstract

Polyarteritis nodosa (PAN) is an immune-mediated necrotizing vasculitis predominantly affecting medium-sized arteries, often manifesting with systemic involvement in its classic form. However, a cutaneous variant (cutaneous polyarteritis nodosa (c-PAN)) confines the disease process to the skin and subcutaneous tissues, typically presenting with painful nodules, chronic ulcerations, and livedo reticularis while sparing major organ systems. Here, we describe a 53-year-old woman with type 2 diabetes and hypertension who developed persistent, painful ulcerative nodules on her lower legs over six months. Laboratory investigations revealed elevated inflammatory markers, negative ANCA, and slightly increased C3. Histopathology confirmed necrotizing arteritis of medium-sized arteries with lobular panniculitis, consistent with c-PAN. She was treated successfully with oral corticosteroids, azathioprine, and supportive wound care, achieving clinical remission without progression to systemic disease. This case underscores the importance of recognizing c-PAN in recalcitrant lower-extremity ulcers and highlights the value of a multidisciplinary approach for optimal patient outcomes.

## Introduction

Polyarteritis nodosa (PAN) is an immune-mediated necrotizing vasculitis that principally affects medium-sized muscular arteries and predominantly affects adults in their fifth decade. The "classic" systemic form of PAN typically involves multiple organ systems, most notably the renal, neurologic, and gastrointestinal systems, and has a male predominance. In contrast, the cutaneous-limited variant (cutaneous polyarteritis nodosa (c-PAN)) tends to affect women more frequently and, while generally confined to the skin and subcutaneous tissue, can still produce significant morbidity through painful subcutaneous nodules, livedo reticularis, and ulcerations [1,2].

The pathophysiology of systemic PAN often involves immune complex deposition, frequently associated with hepatitis B virus infection, leading to complement activation and endothelial damage. Conversely, in c-PAN, some studies have identified autoantibodies similar to those seen in antiphospholipid syndrome, suggesting distinct immunologic mechanisms [1].

Given its relative rarity and an often-overlapping clinical spectrum with conditions such as livedoid vasculopathy or other medium-to-small vessel vasculitides, diagnosing c-PAN depends on a combination of thorough systemic evaluations and characteristic histopathologic findings [3]. Herein, we report a chronic and successfully managed case of c-PAN in a middle-aged patient with metabolic comorbidities, highlighting the diagnostic considerations and therapeutic outcomes.

## Case presentation

A 53-year-old married woman with a five-year history of type 2 diabetes and one year of hypertension presented with painful, progressively enlarging nodules and ulcerations on both lower legs. These lesions persisted for six months, intermittently healing and then recurring, leading to considerable discomfort and functional limitation. She reported occasional paresthesia in her feet, arthralgias, and two first-trimester miscarriages over a decade earlier, but no additional obstetric or gynecological issues.

**Figure 1:**
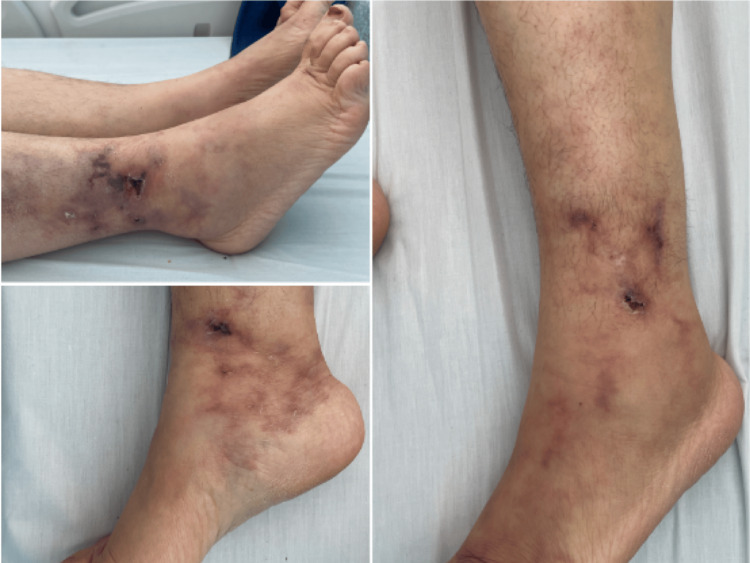
Perimalleolar ulcerations and branching livedoid pattern on the lower legs Upper left panel showing the ulceration and livedo reticularis on the external side of the right leg. Lower right panel showing a smaller ulceration and livedo reticularis on the medial side of the left ankle. Panel on the right showing branched livedo and ulceration of the external side of the left leg.

Physical examination revealed multiple tender, round-to-irregular subcutaneous nodules that sometimes ulcerated, forming necrotic or hemorrhagic crusts, predominantly located in the perimalleolar region and the lower third of both legs. Livedo reticularis was noted, along with areas of hyperpigmentation (Figure 1).

Laboratory tests demonstrated elevated inflammatory markers (erythrocyte sedimentation rate (ESR) up to 150 mm/h, C-reactive protein (CRP) at 45 mg/L), borderline high C3 with normal C4, and negative serologies for hepatitis B/C, HIV, parvovirus B19, ANCA, and cryoglobulins (Table 1).

**Table 1:** The patient's laboratory test results CRP: C-reactive protein; ESR: erythrocyte sedimentation rate

Laboratory tests	Value	Reference range
C3	1.89 g/L (slightly elevated, indicating inflammation)	0.75 - 1.75 g/L
C4	0.38 g/L	0.2 - 0.5 g/L
ESR	150 mm/h (markedly elevated, indicating inflammation)	0-15 mm/hr
CRP	45 mg/L (markedly elevated, indicating inflammation)	10 mg/L
Hepatitis B serology	Negative (no significant level of detectable)	Negative
Hepatitis C antibody	Negative (no significant level of detectable)	Negative
HIV serology	Negative (no significant level of detectable)	Negative
ANCA	Negative (no significant level of detectable)	< 20
Cryoglobulins	Negative (no significant level of detectable)	Negative
Parvovirus B19	Negative (no significant level of detectable)	< 0,9 IV

**Figure 2:**
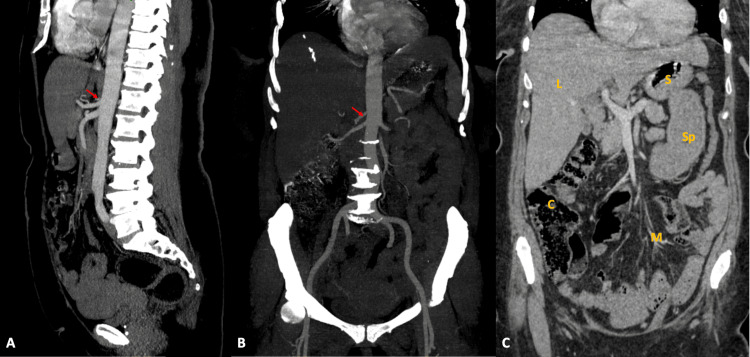
Abdominal angioscan Sagittal (A) and coronal (B) angioscanner reconstruction showing good opacification of the abdominal aorta (red arrows), its various visceral branches, and the iliac arterial axes, with a normal caliber and parallel walls, with no atheromatous, inflammatory, or additive parietal thickening. Coronal reconstruction at portal time (C) of abdominal scanner showing no digestive parietal thickening or other visceral abnormality. S: stomach; L: liver; C: colon; M: mesentery; Sp: spleen

Imaging studies (Doppler ultrasound, abdominal angioscan) showed no arterial stenosis, aneurysms, or visceral lesions (Figure 2).

**Figure 3:**
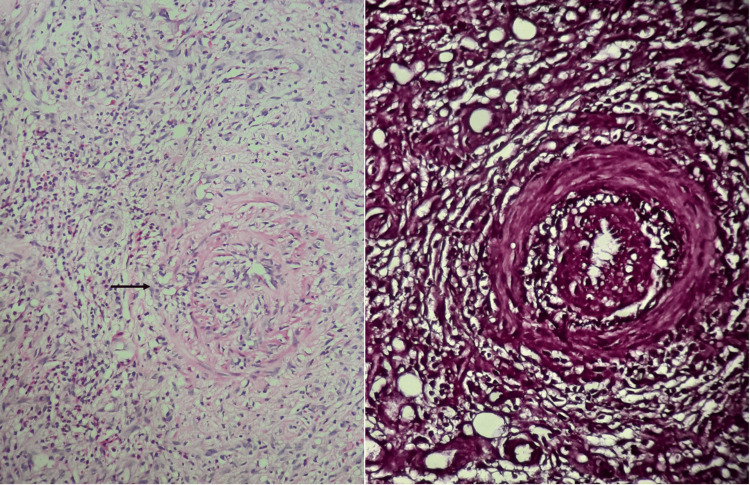
Histologic image of the deep incisional biopsy taken at the edge of an ulcer Left image showing inflammatory infiltrate around medium-caliber vessels with leukocytoclasis and fibrinoid necrosis, giving an “onion-bulb” appearance (black arrow, hematoxylin, eosin, and saffron (HES) ×200). Image on the right of orcein staining highlighting the elastic fibers of an artery (orcein x200).

A deep incisional biopsy at the edge of a representative ulcer confirmed necrotizing inflammation in medium-sized arterial walls with lobular panniculitis, consistent with c-PAN (Figure 3).

Electromyography indicated mild peripheral neuropathy, primarily attributed to her diabetes.

**Figure 4:**
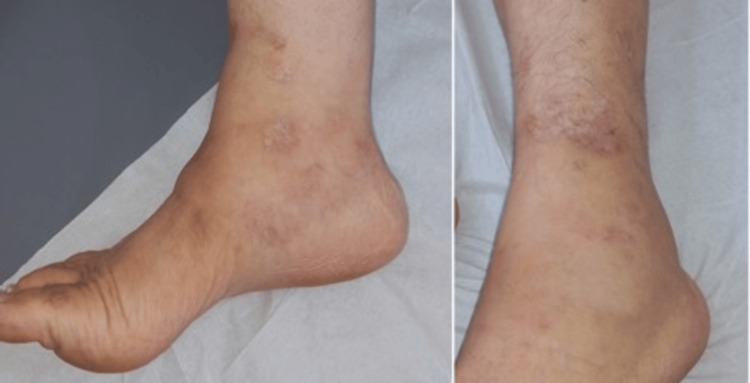
Post-treatment follow-up with disappearance of livedo and subcutaneous nodules and residual atrophic whitish scarring. The left panel corresponds to the right lower leg and the right panel represents the left lower leg.

She began oral prednisone (0.5 mg/kg/day) and low-dose aspirin, later adding azathioprine as a steroid-sparing agent (200 mg/day). Daily wound care included debridement and dressing changes, which accelerated ulcer healing. Improvement in symptoms and noticeable wound healing were observed one month after treatment initiation. Over the ensuing months, prednisone was gradually tapered as lesions improved and discontinued by six months. Azathioprine was then reduced to 100 mg/day and completely stopped after one year without experiencing relapse or systemic progression (Figure 4).

## Discussion

c-PAN is a variant of PAN confined to the skin and subcutaneous tissues, sparing major organ systems in most cases [1,4]. Like systemic PAN, c-PAN is characterized by segmental necrotizing inflammation of medium-sized muscular arteries, which can lead to ischemia and infarction of the affected tissues. However, distinct from the classic systemic form, patients with c-PAN typically present with chronic or relapsing painful nodules, ulcerations, and livedo reticularis but lack systemic features such as significant renal, neurological, or gastrointestinal involvement [2]. 

Our patient’s profile (middle-aged, female, with comorbidities (type 2 diabetes and hypertension)) added complexity to wound healing and heightened the initial suspicion for alternative diagnoses like livedoid vasculopathy or necrotic angiodermatitis. The main distinctive features of these differential diagnoses are discussed in Table 2.

**Table 2:** Clinical, histopathological, and evolutive features of cutaneous polyarteritis nodosa (c-PAN), livedoid vasculopathy, and necrotic angiodermatitis. Adapted from [5-7].

	Cutaneous polyarteritis nodosa (c-PAN)	Livedoid vasculopathy	Necrotic angiodermatitis
Epidemiology	Women in the age group of 45 to 50 years old	Women who are 45 to 50 years old with a history of diabetes and arterial hypertension	Women in the age group of 50 to 70 years with cardiovascular comorbidities
Common clinical presentation	Ramified livedo, purpura, necrosis, and ulceration with symmetrical involvement of the lower 1/3 of the legs, associated with white atrophy. No signs of venous insufficiency. No systemic involvement		Necrotic ulcer on the posterolateral legs.
Distinct clinical findings	Subcutaneous nodules along arterial vessels, and loco-regional peripheral neuropathy	Nociceptive pain	Ragged and purpuric margins, rapidly extensive, and associated with exquisite pain
Histopathology	Segmental fibrinoid necrosis of medium-calibre arteries with perivascular neutrophilic infiltrate	Thrombosis of vessels in the superficial or middle dermis, with segmental hyalinization and lymphocytic perivascular infiltrate	Subcutaneous arteriosclerosis with hypertrophy of the media, thickening of the lamina, and intimal hyperplasia
Evolution	Chronic, relapsing-remitting course		Chronic, with no tendency to wound healing

The negative ANCA in our patient, normal or only slightly altered complements, and absence of hepatic viral serologies also narrowed possibilities toward a vasculitis of medium-sized vessels rather than a small-vessel process. Finally, the key element was the histopathologic confirmation showing fibrinoid necrosis and perivascular infiltrates in medium-sized arteries within the subcutaneous fat [2, 3]. 

Long-term management of c-PAN typically involves corticosteroids as first-line therapy, often in conjunction with steroid-sparing agents such as azathioprine or methotrexate [1, 3, 8, 9]. This approach helps mitigate the side effects of prolonged high-dose steroid use. In our case, close follow-up ensured no systemic manifestations emerged, validating the diagnosis of a purely cutaneous-limited vasculitis. Recent studies have shown that remission and prevention of relapse can be achieved in many c-PAN cases if early, aggressive treatment is introduced, particularly in patients with comorbidities that might predispose them to worse outcomes [4, 10]. 

Although c-PAN is classically benign compared to systemic PAN, systemic evolution can occur in a small subset of patients, underscoring the need for routine monitoring, including laboratory screenings and clinical evaluations for evolving organ involvement [2]. The patient in this report highlights how a diligent, multidisciplinary care model, encompassing dermatology, rheumatology, and endocrinology, can optimize results. Key findings in our case include clinical presentation, absence of systemic involvement, histopathologic evidence, and favorable response to combined immunosuppressive and supportive therapies, with no relapse after gradual tapering. 

Clinically, her outcome underscores the importance of early recognition and the role of a combined therapeutic strategy for sustained remission. The case also reaffirms that metabolic and vascular risk factors (diabetes, hypertension) may complicate the clinical picture by delaying ulcer healing, demanding a holistic approach to optimize comorbidity management.

## Conclusions

c-PAN should be strongly considered in patients presenting with persistent, painful nodules and ulcerations confined to the lower extremities, particularly when small-vessel vasculitis and other vasculopathies have been excluded. Confirmatory biopsy, comprehensive systemic evaluation, and timely initiation of immunosuppressive therapy are crucial. This case illustrates the potential for complete remission and prevention of systemic progression with appropriate treatment and highlights the possibility of other differential diagnoses of chronic necrotic ulcerations of the lower legs in a middle-aged woman.
